# Supplementary material for: Curcumin improves atrial fibrillation susceptibility by regulating tsRNA expression in aging mouse atrium
Source: PeerJ. 2024 Jul 26;12:e17495. doi: 10.7717/peerj.17495 (PMC11285363; doi:10.7717/peerj.17495)
Supplement: Supplemental Information 9 [file peerj-12-17495-s009.docx]

NOTE: Please save this file locally before filling in the table, DO NOT work on the file within your internet browser as changes will not be saved. Adobe Acrobat Reader (available free [here](https://acrobat.adobe.com/uk/en/acrobat/pdf-reader.html)) is recommended for completion.


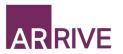


The ARRIVE guidelines 2.0: author checklist

The ARRIVE Essential 10

These items are the basic minimum to include in a manuscript. Without this information, readers and reviewers cannot assess the reliability of the findings.

**Item**

**Recommendation**

**Section/line**

**number,** **or** **reason**

**for** **not** **reporting**

| **Study** **design** | 1 | For each experiment, provide brief details of study design including:  a. The groups being compared, including control groups. If no control group has been used, the rationale should be stated.  b. The experimental unit (e.g. a single animal, litter, or cage of animals). | Methods/Line108-110  Methods/Line 109 |
| --- | --- | --- | --- |
| **Sample** **size** | 2 | a. Specify the exact number of experimental units allocated to each group, and the total number in each experiment. Also indicate the total number of animals used.  b. Explain how the sample size was decided. Provide details of any *a* *priori* sample size calculation, if done. | Methods/Line 109  Each experiment was repeated three times to count the number of experimental ani  mal |
| **Inclusion** **and** **exclusion** **criteria** | 3 | a. Describe any criteria used for including and excluding animals (or experimental units) during the experiment, and data points during the analysis. Specify if these criteria were established *a* *priori.* If no criteria were set, state this explicitly.  b. For each experimental group, report any animals, experimental units or data points not included in the analysis and explain why. If there were no exclusions, state so.  c. For each analysis, report the exact value of *n* in each experimental group. | Methods/Line 102-108  There were no ex cluded  Methods/Line 109 |
| **Randomisation** | 4 | a. State whether randomisation was used to allocate experimental units to control and treatment groups. If done, provide the method used to generate the randomisation sequence.  b. Describe the strategy used to minimise potential confounders such as the order of treatments and measurements, or animal/cage location. If confounders were not controlled, state this explicitly. | N/A.The Randomisation  Sequence was not  involved in this study  N/A.The Randomisation  Sequence was not  involved in this study |
| **Blinding** | 5 | Describe who was aware of the group allocation at the different stages of the experiment (during the allocation, the conduct of the experiment, the outcome assessment, and the data analysis). | NA. No double or triple blindness in clinical trial s  was involved in this study |
| **Outcome**  **measures** | 6 | a. Clearly define all outcome measures assessed (e.g. cell death, molecular markers, or behavioural changes).  b. For hypothesis-testing studies, specify the primary outcome measure, i.e. the outcome measure that was used to determine the sample size. | Methods/Line214-297  This paper does not address the hypothesis testing stud ie |
| **Statistical**  **methods** | 7 | a. Provide details of the statistical methods used for each analysis, including software used.  b. Describe any methods used to assess whether the data met the assumptions of the statistical approach, and what was done if the assumptions were not met. | Methods/Line210-213  This paper does not address the hypothesis testing stud ie |
| **Experimental** **animals** | 8 | a. Provide species-appropriate details of the animals used, including species, strain and substrain, sex, age or developmental stage, and, if relevant, weight.  b. Provide further relevant information on the provenance of animals, health/immune status, genetic modification status, genotype, and any previous procedures. | Methods/Line106-111  Methods/Line106-107 |
| **Experimental** **procedures** | 9 | For each experimental group, including controls, describe the procedures in enough detail to allow others to replicate them, including:  a. What was done, how it was done and what was used.  b. When and how often.  c. Where (including detail of any acclimatisation periods).  d. Why (provide rationale for procedures). | Methods/Line101-207  Methods/Line101-207  Methods/Line101-207  It was designed according t o relevant  basic experimental method |
| **Results** | 10 | For each experiment conducted, including independent replications, report:  a. Summary/descriptive statistics for each experimental group, with a measure of variability where applicable (e.g. mean and SD, or median and range).  b. If applicable, the effect size with a confidence interval. | Methods/Line 214-297  Methods/Line 214-297 |


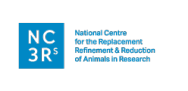


The Recommended Set

These items complement the Essential 10 and add important context to the study. Reporting the items in both sets represents best practice.

**Item**

**Recommendation**

**Section/line**

**number,** **or** **reason**

**for** **not** **reporting**

| **Abstract** | 11 | Provide an accurate summary of the research objectives, animal species, strain and sex, key methods, principal findings, and study conclusions. | Abstract/Line26-45 |
| --- | --- | --- | --- |
| **Background** | 12 | a. Include sufficient scientific background to understand the rationale and context for the study, and explain the experimental approach.  b. Explain how the animal species and model used address the scientific objectives and, where appropriate, the relevance to human biology. | Abstract/Line 48-98  Abstract/Line 48-98 |
| **Objectives** | 13 | Clearly describe the research question, research objectives and, where appropriate, specific hypotheses being tested. | Abstract/Line91-98 |
| **Ethical**  **statement** | 14 | Provide the name of the ethical review committee or equivalent that has approved the use of animals in this study, and any relevant licence or protocol numbers (if applicable). If ethical approval was not sought or granted, provide a justification. | Ethics declar ations/Line102-106 |
| **Housing** **and**  **husbandry** | 15 | Provide details of housing and husbandry conditions, including any environmental enrichment. | Methods/Line106-108 |
| **Animal** **care** **and** **monitoring** | 16 | a. Describe any interventions or steps taken in the experimental protocols to reduce pain, suffering and distress.  b. Report any expected or unexpected adverse events.  c. Describe the humane endpoints established for the study, the signs that were monitored and the frequency of monitoring. If the study did not have humane endpoints, state this. | Methods/Line 111-112  N/A.  Methods/Line 128-129 |
| **Interpretation/** **scientific** **implications** | 17 | a. Interpret the results, taking into account the study objectives and hypotheses, current theory and other relevant studies in the literature.  b. Comment on the study limitations including potential sources of bias, limitations of the animal model, and imprecision associated with the results. | Discussion/Li ne200-390  Discussion/Li ne391-395 |
| **Generalisability/** **translation** | 18 | Comment on whether, and how, the findings of this study are likely to generalise to other species or experimental conditions, including any relevance to human biology (where appropriate). | No other species were studied in depth |
| **Protocol**  **registration** | 19 | Provide a statement indicating whether a protocol (including the research question, key design features, and analysis plan) was prepared before the study, and if and where this protocol was registered. | A protocol was prepared before the study without registration |
| **Data** **access** | 20 | Provide a statement describing if and where study data are available. | Data Sharing statement |
| **Declaration** **of**  **interests** | 21 | a. Declare any potential conflicts of interest, including financial and non-financial. If none exist, this should be stated.  b. List all funding sources (including grant identifier) and the role of the funder(s) in the design, analysis and reporting of the study. | Competing intere sts/Line401-402  Acknowledgments/Lin e 419-423 |

Please leave this space alone as it will be supplemented by the editorial office when needed.

[www.ARRIVEguidelines.org](http://www.arriveguidelines.org)
